# SARS-CoV-2 infection and SLE: endothelial dysfunction, atherosclerosis, and thrombosis

**DOI:** 10.1007/s10067-022-06497-1

**Published:** 2023-01-09

**Authors:** Wojciech Płazak, Leszek Drabik

**Affiliations:** 1 Department of Cardiac and Vascular Diseases, John Paul II Hospital, Jagiellonian University Medical College, Krakow, Poland; 2 Department of Pharmacology, Jagiellonian University Medical College, Krakow, Poland

**Keywords:** Autoantibodies, Endothelium, Lupus erythematosus, Rheumatic diseases, SARS-CoV-2; Thrombosis

## Abstract

An increased risk of atherosclerotic and thrombotic complications characterizes connective tissue diseases. Endothelial dysfunction is the basis for the initiation and progression of atherosclerosis and thrombosis. We present systemic lupus erythematosus (SLE) as a model rheumatic disease with endothelial dysfunction and discuss its mechanisms, factors that influence the early onset and rapid progression of atherosclerosis, and the increased risk of thromboembolic events. We focus on established methods to improve endothelium function, including statins, antiplatelet, and antithrombotic therapy. Hypercoagulable and hypofibrinolitic states and a hyperinflammatory response characterize severe acute respiratory syndrome coronavirus 2 (SARS-CoV-2) infection. Several pathogenic mechanisms are typical for an acute phase of Covid-19 post-Covid syndrome and connective tissue diseases: endothelial dysfunction, elevated antiphospholipid antibody titer, activation of the complement system, and formation of extracellular neutrophil traps (NET). The current review discusses the mechanisms underlying SLE and the COVID-19 in the context of endothelial function, atherosclerosis, and thrombosis (Graphical abstract).

**Table:** 

**Key Points** *• The pathophysiology of systemic lupus erythematosus (SLE) and Covid-19 shows some similarities, such as endothelial cell activation and dysfunction, the activation of complementary systems, the presence of antiphospholipid antibodies, and the formation of extracellular neutrophil traps.* *• Autoimmunity in both diseases creates the basis for hyperinflammatory, hypercoagulable, and hypofibrinolitic states and their thromboembolic complications.* *• This paper presents our perspective on the mechanisms behind the cardiovascular manifestations of SLE and COVID-19, with a particular emphasis on endothelial dysfunction.*

**Graphical abstract:**

Covid-19 and systemic lupus erythematosus—potential similarities in pathophysiology. Figures of the panel illustrate the clinical manifestations of endothelial dysfunction, atherosclerosis, and thromboembolism, including coronary artery disease ([A] coronary angiography with left anterior descending artery stenosis and [B] scintigraphy with reduced perfusion in the myocardial apical segments), stroke ([C] carotid angiography, left carotid artery occlusion) and pulmonary embolism ([D]computed tomography with thrombus in the right pulmonary artery). 
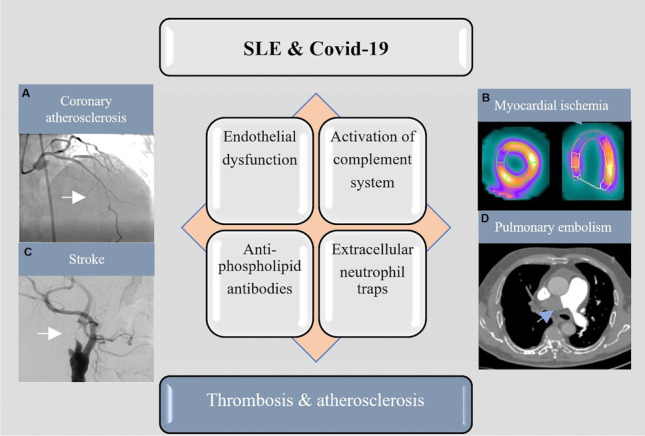

## Introduction

Connective tissue diseases were defined as a separate group in 1941 as systemic pathology with a wide range of clinical symptoms, but with similar histopathological changes based on fibrillar necrosis of the connective tissue [1]. We may now include in this group systemic lupus erythematosus (SLE), systemic sclerosis, dermatopolymyositis, rheumatoid arthritis (RA), and systemic vasculitis.

Urowitz et al. [2] observed in 1976 that the frequent cause of death in SLE patients suffering from the disease for more than a year was myocardial infarction, but not the direct consequences of autoimmunity. Further research has shown that one of the most important prognostic factors in SLE is heart pathology caused by the rapid development of coronary artery atherosclerosis and thrombosis, and emboli of the heart vessels. In the era of steroid therapy, hemodynamically significant endocardial morphologic changes (especially heart valve leaflets) decreased, but the problem of cardiovascular incidences caused by atherosclerosis remained. It is noteworthy that steroids, in a healthy heart and SLE, increase the amount of fatty tissue in the heart, stimulate muscle hypertrophy, and accelerate atherosclerosis [3]. In published studies, the percentage of cardiovascular deaths in SLE patients (mainly due to myocardial infarction) was as high as 40 [4, 5]. The risk of myocardial infarction in women with SLE aged 35 to 45 years is 50 times higher than in the general population [6]. In most cases, coronary atherosclerosis develops subclinically and the first symptom may be myocardial infarction [6, 7]

## SLE and endothelial dysfunction

These data led to the research’s interest to vascular endothelium in SLE and other rheumatic diseases: Endothelial dysfunction forms a ground for atherosclerosis onset and progression, as well as thrombosis. Furthermore, endothelial dysfunction may be considered a local inflammation directly related to general inflammation in rheumatic diseases. During the inflammatory process, the phenotype of endothelial cells becomes activated [8]. Nuclear transcription factor-κB (NF-κB) regulates the expression of adhesion molecules, such as intercellular adhesion molecule-1 (ICAM-1), vascular cell adhesion molecule-1 (VCAM-1), and E-selectin that play a pivotal role in leucocyte-endothelium interactions [8].

Several mechanisms have been proposed to understand endothelial dysfunction in rheumatic diseases. Impaired clearance of apoptotic cells, oxidative stress, activation of B cells with different circulation autoantibodies, subtypes of T lymphocytes or cascade of cytokines [9], or monocyte stimulation [10] have been proposed as the main pathogenic way. Recently, the role of anti-endothelial cell antibodies has also been suggested [11]. Furthermore, circulating endothelial cells were associated with thromboembolic events in patients with antiphospholipid antibodies [12].

Endothelial dysfunction with abnormal vascular reactivity was shown in pediatric-onset SLE patients [13] and adult-onset SLE patients, although they were treated with modern protocols [13, 14]. Endothelial dysfunction is present in patients with SLE that are naive to cardiovascular diseases, and diabetes mellitus, renal disease, or hypertension are only additional contributors [15].

As stated above, the most important clinical features of endothelial dysfunction are the onset and progression of atherosclerosis, together with vascular thrombosis.

## SLE and early onset atherosclerosis

**Fig. 1:**
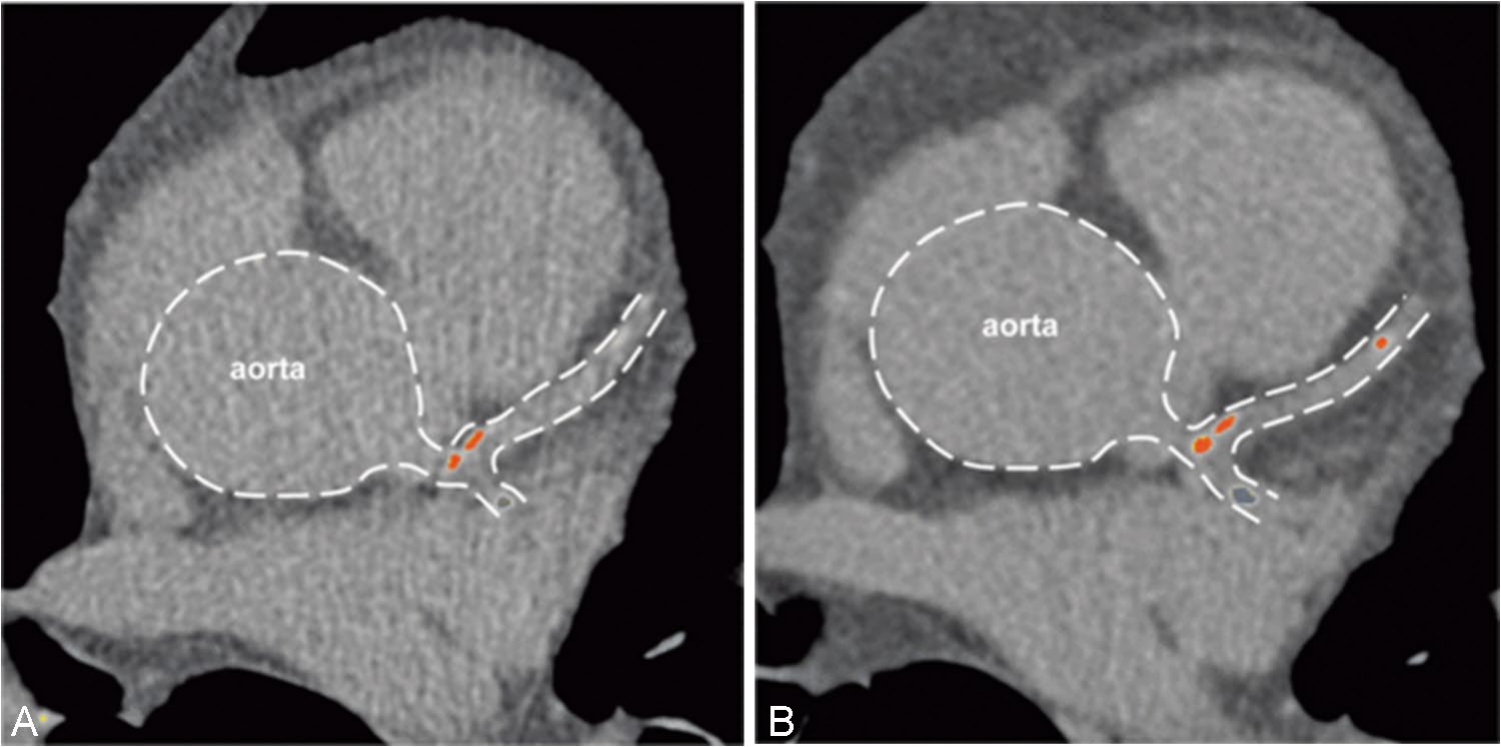
Progression of atherosclerosis in an SLE patient with no cardiovascular complaints at 1 year of follow-up. Multidetector CT calcium score examination. **A** Two calcified plaques are seen in the left anterior descending artery (red) and one calcified plaque in the circumflex artery (blue): plaque volume 156.4 mm^3^, calcium score 138.9. **B** After 1 year, the volume of the previously observed plaques increased with the new calcification in the distal part of the left anterior descending artery: plaque volume 223 mm^3^, calcium score 202.5

Image studies showed that coronary atherosclerosis develops rapidly in young patients despite the stable stage of SLE and maintenance therapy with low doses of steroids [16]. Figure 1 shows the progression of coronary atherosclerosis seen on multidetector computed tomography (CT) in a patient with SLE without cardiovascular complications at a 1-year follow-up.

Atherosclerotic plaques in the arteries are detected in almost half of young asymptomatic SLE patients. The most frequently affected are the coronary arteries (42% of patients with calcifications seen on CT) and the carotid arteries (24%) [17].

The high risk of symptomatic ischemic heart disease in young patients with SLE shows that the classical risk factors for atherosclerosis do not constitute the main etiological factor in this group. According to expectations, no significant influence of obesity, arterial hypertension, smoking, hypercholesterolemia, or diabetes on the presence of atherosclerosis or myocardial perfusion was found in young patients with SLE [7]. General inflammation manifested by an increase in the level of C-reactive protein (CRP) and the decrease in complement C3c and C4 levels also does not intensify the progression of atherosclerosis in young people [7] if only CRP does not permanently increase to at least 20 mg/l [18].

In SLE patients with myocardial perfusion defects or atherosclerotic plaques detected in CT-derived calcium score, high autoimmunity was proved, manifested by an increased level of antiphospholipid antibodies, mainly anticardiolipin (aCL) IgG and anti-β_2_-glycoprotein I (antiβ_2_GPI) IgG antibodies [7, 19]. These antibodies may initiate and accelerate lipid deposition and plaque formation [20]. In in vitro studies, antiβ_2_GPI antibodies were shown to accelerate the binding of aCL to endothelial cells, leading to thrombosis [21]; antiβ_2_GPI may also bind directly to oxidized low-density lipoprotein (LDL), forming highly immunogenic complexes [21]. On the other hand, in young patients under 45 years of age, with premature ischemic heart disease, who underwent myocardial revascularization, aPL levels were significantly higher than in healthy young subjects [22]. More recent studies show a significant association between the IgM class of anticardiolipin and antiβ2GPI antibodies with vascular endothelial activation and prothrombotic status of patients [16]. Substantially higher ICAM-1 concentration, indicating increased vascular endothelial activation, observed in patients with SLE, is correlated with elevated levels of IgM class antiphospholipid antibodies (aCL IgM > 30 MPL and β_2_GPI IgM > 20 SMU). Interestingly, endothelial activation, also associated with serum markers of the inflammatory process of SLE (low C4; increased CRP or IL-6), appears not to be associated with the SLE activity index (Systemic Lupus Erythematosus Disease Activity Index [SLEDAI]) [23]. Interactions between platelets and vascular endothelial cells are believed to occur in atherosclerosis, with increased expression of adhesion molecules and their ligands [24–27]. Activation of CD40 in vascular endothelial cells results in increased expression of ICAM on their surface, increasing the instability of atherosclerotic plaques in the coronary arteries, increasing the risk of their rupture, and thus initiation of thrombotic process clinically manifesting itself as unstable angina and even sudden cardiac death [28, 29]. In patients with SLE aPL-positive with clinical episodes of thrombosis, soluble CD40L is elevated [30]. Therefore, increased ICAM-1 levels may be a marker of the severity of the atherosclerotic process [31–33].

Several studies suggest that, in addition to the role of type I interferons (INFs) in the pathogenesis of lupus, they may be important contributors to premature atherosclerosis in SLE [34]. Type I INFs promote an antiangiogenic signature, foam cell formation, and platelet activation [35].

Elevated concentrations of von Willebrand factor (vWF) are another marker of endothelial activation and damage [36]. Increased vWF is associated with the risk of thrombotic events and possibly coronary heart disease [37]. However, contrasting opinions are presented on whether elevated vWF values influence the progression of the atherosclerotic process [38, 39]; even claims negate this association [40]. It has also been suggested that vWF is not causally related to atherosclerosis, but rather that the development of atherosclerosis leads to elevated plasma vWF, which favors arterial thrombosis [41]. A reliable assessment is hampered by the fact that many nonspecific factors, such as age, hyperlipidemia, and hypertension, affect the concentration of vWF in plasma [42]. Furthermore, the methodology for measuring the vWF concentration is not fully standardized.

Recently, an increased risk of thromboembolic complications associated with autoimmune diseases, such as SLE, outside of the context of antiphospholipid syndrome, has been documented [43]. Plasma thrombin-antithrombin complex (TAT) concentrations represent a short-lived marker of this prothrombotic tendency. Inflammation and thrombosis processes are interconnected, and an association between elevated CRP and IL-6 values with the thromboembolic process was also shown in the literature [44]. Elevated plasma TAT concentrations were observed in patients with elevated levels of aCL IgM (> 30 MPL) [23]. However, recent analyses show that antiphospholipid antibodies of the IgG class, and not of the IgM class, are generally associated with venous and arterial thromboembolic in patients with SLE [43, 45]. Nevertheless, the clinical significance of IgM class aPL in antiphospholipid syndrome (APS) has also been documented [46]. We may speculate that elevated levels of the aCL IgM class may appear as an early marker that influences the risk of future thromboembolic events, and then the IgG class follows, as described in the literature, serving as the late marker of chronic vascular pathologies.

The measurement of D-dimers is the screening test for thromboembolic events in everyday practice. However, the assessment of D-dimers is characterized by low specificity: Their elevated levels are often present in hospitalized patients, particularly in the elderly, in people with cancer, after recent surgical procedures, in the course of renal failure, and in many other conditions, including the second and third trimesters of normal pregnancy [47–49].

The correlation between elevated levels of IgM class antiphospholipid antibodies and two factors that may enhance atherosclerosis, endothelial activation/damage (ICAM) and prothrombotic stage (TAT), may be of great importance. SLE patients are classified as being in remission according to disease activity indexes (e.g., SLEDAI), in which low complement or increased DNA binding are the parameters included in the assessment. Antiphospholipid antibody levels are not included in these scales, although they may contribute to the gradual progression of atherosclerosis and, as a result, the prognosis of patients with SLE.

In patients with coronary calcifications, higher antinuclear antibody titers were also detected [7]. Only a few papers have been published on the possible atherogenic action of antinuclear antibodies (ANA). In vitro studies, immune complexes composed of anti-dsDNA, DNA, and LDL lead to increased cholesterol deposition in the artery walls and reveal cytotoxic action [50]. ANA have been shown to have a prognostic value for the development of clinically significant ischemic heart disease, even in people without autoimmune disease [51].

A high level of antiphospholipid antibodies may influence pathological changes in heart valve leaflets [52, 53]. In more than 30% of patients with SLE with high concentrations of aPL IgG concentration (> 80 IU/ml), the nodules are observed in valve leaflets and the frequency of this pathology decreases with lower levels of aPL IgG levels (16–80 IU/ml, 20% of patients) and in patients without aPL IgG (4% of patients) [52]. The pathology of heart valve leaflets correlates with the general intensity of inflammation manifested by an increase in the CRP level and the levels of the C3c and C4 components of the complement system decrease [7]. Complement system activation has also been shown to enhance atherosclerosis progression [54].

The activation or damage of the vascular endothelium is manifested by the elevation of ICAM-1 and vWF [55, 56]. In patients with coronary calcifications, the levels of these molecules are significantly higher compared to those without calcium deposits in the coronary arteries [57]. Furthermore, the connection between the presence of calcified coronary plaque and the levels of selectin E, VCAM, or tumor necrosis factor-alpha (TNFα) levels was shown [55].

## SLE and thromboembolism

Feinstein and Rapaport used the term “lupus anticoagulant” (LA) for the first time in 1972 [58] as an in vitro coagulation inhibitor in patients with SLE. Further research revealed that in vivo LA may cause thrombosis. The term “antiphospholipid syndrome” was implemented in 1987 [59, 60], with the following laboratory criteria: LA presence (positive twice on a distance of at least 12 weeks) together with aCL or anti-ß_2_GPI [61].

Although antiphospholipid syndrome has been known for 35 years, the exact mechanism of clot formation is not well understood. The reaction of antiphospholipid antibodies with C protein and components of the complement system is postulated, with a decrease in the protective anticoagulation role of annexin A5, activation of platelets, monocytes, and endothelial cells, which change their phenotype to procoagulant [62]. In patients with high aPL titers, endothelial damage leads to significantly higher thrombin generation than in patients without aPL [63]. The antibacterial plasma protein ß_2_GPI increases phagocytosis of phospholipid-exposing microparticles and apoptotic cells, inhibits platelet adhesion and aggregation mediated by vWF, and prevents protein S inactivation by the C4b-binding protein. These antithrombotic functions of ß_2_GPI are affected by antiß_2_GPI antibodies [64]. Furthermore, ß_2_GPI antibody complexes bind to cellular receptors on endothelial cells, monocytes, neutrophils, and platelets, activating these cells and enhancing their thrombogenicity [64].

**Fig. 2:**
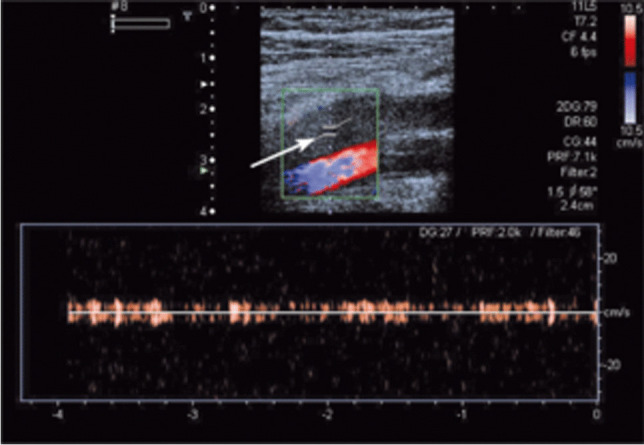
Femoral vein thrombosis in a patient with antiphospholipid syndrome. There is no flow in the vein (arrow), with normal flow in the artery (below)

The most frequent clinical manifestation of antiphospholipid syndrome is venous thrombosis, especially in the deep veins of the lower extremities (Fig. 2).

**Fig. 3:**
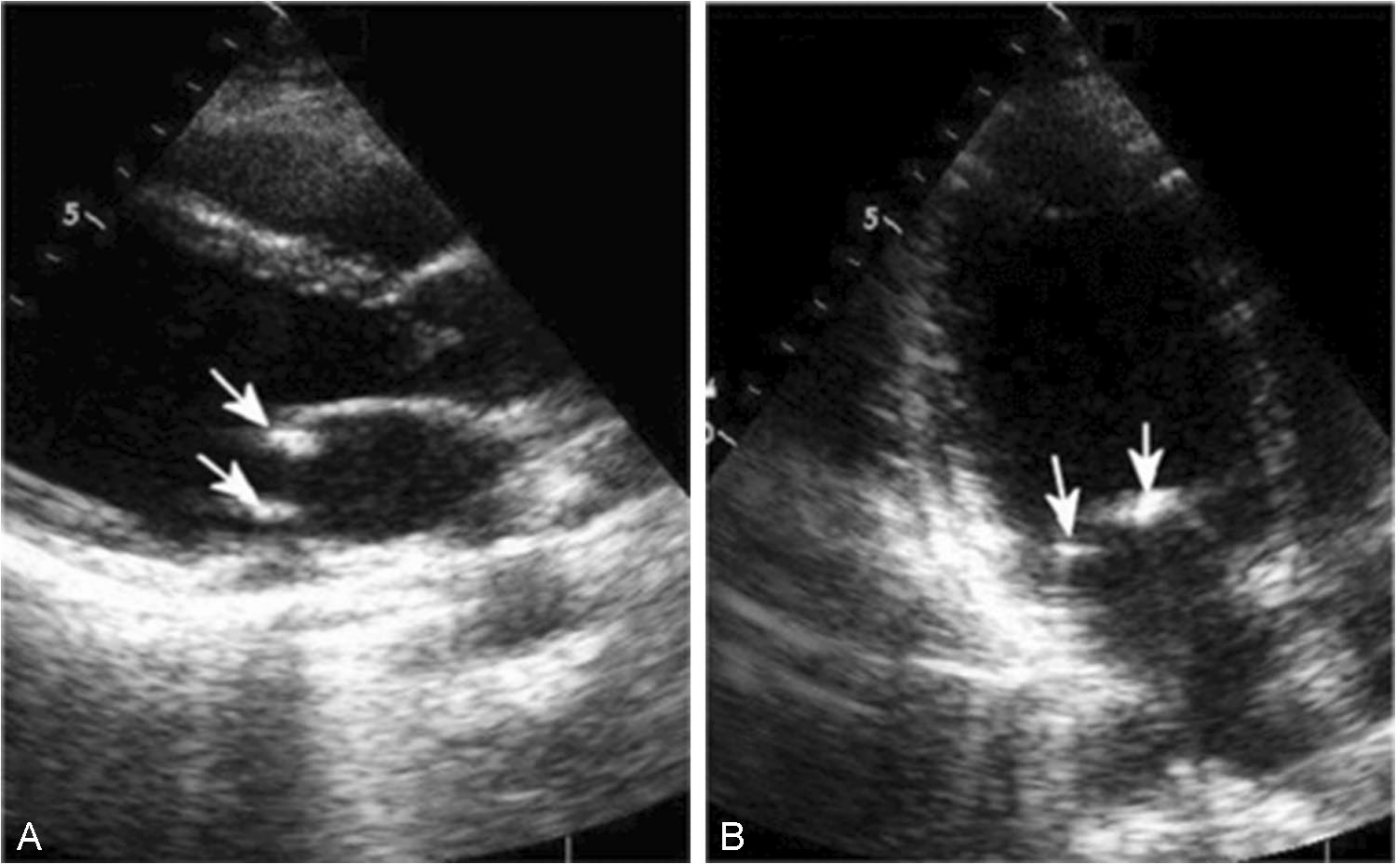
A young patient with antiphospholipid syndrome and ischemic stroke at the age of 21 years. Mitral leaflets are thickened with round nodules at the edges seen in the parasternal (**A**) and apical (**B**) echocardiographic window. Nodules may serve as the base for clot formation and increase the risk of embolization

Arterial thrombosis is rare and is detected mainly in the brain arteries (Fig. 3) but may be present in the coronary and other arteries [65].

**Fig. 4:**
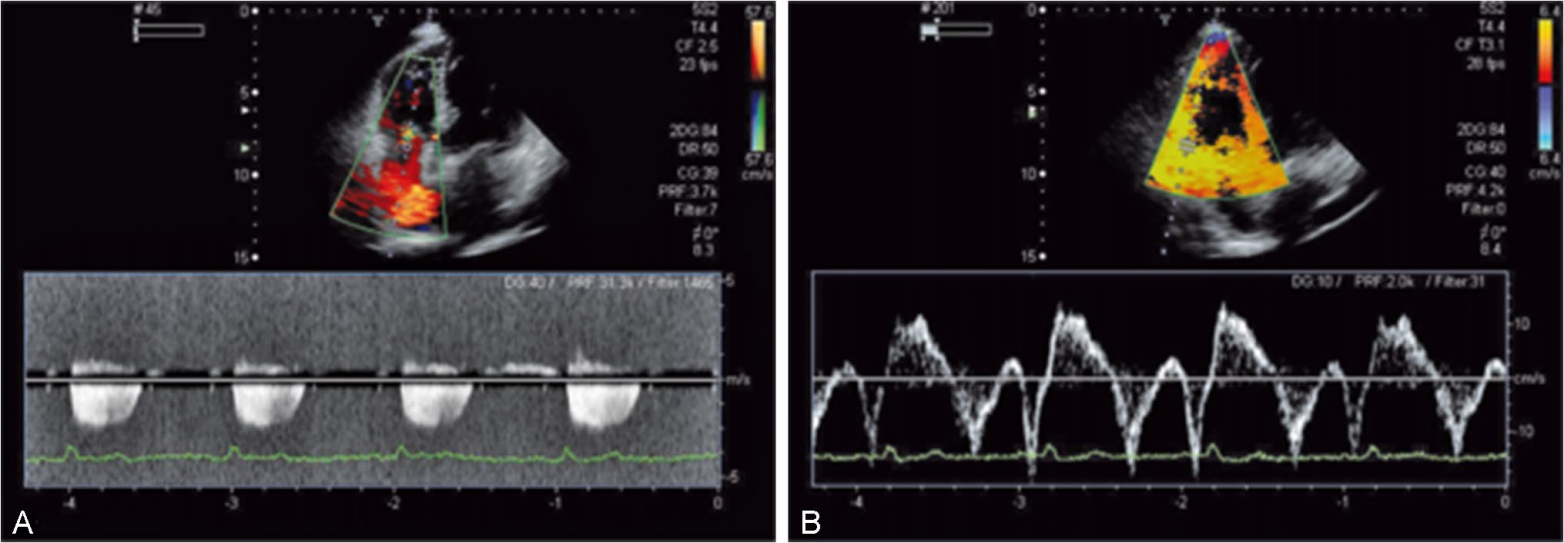
**A** Elevated right ventricle systolic pressure in SLE patient and high levels of aCL IgG (26.11 RU/ml) and antiß_2_GPI IgG (3.66 RU/ml). The risk of pulmonary hypertension in SLE increases when aCL IgG > 20 RU/ml, antyß_2_GPI IgG > 3 RU/ml [62]. The tricuspid regurgitation gradient is 27 mmHg, the systolic pressure of the right ventricle 37 mmHg. **B** Despite increased RV systolic pressure, the function of the right ventricle remains: on examination of TDE, the velocity of the tricuspid annulus in systole (13 cm/s) and early diastole (18 cm/s) is normal

In patients with antiphospholipid syndrome after the first thromboembolic episode, the risk of the new episode is especially high if LA is present together with high IgG aCL titers, as shown in the meta-analysis of 25 studies [66], or in triple positive patients (LA + IgG aCL + IgG antiß_2_GPI) [67, 68]. In addition to clinically seen vascular thrombotic episodes, microthrombosis may form a substantial prognostic factor. Increased antiß2GPI titers for aCL or IgG were shown to correlate with the right ventricle systolic pressure (Fig. 4) [68].

SLE patients with diagnosed pulmonary hypertension have higher frequency of aCL positivity than in SLE people with normal pulmonary pressure [69]. In mixed connective tissue disease (MCTD), pulmonary hypertension was shown to be related to antiß_2_GPI levels [70]. Higher systolic pulmonary pressure (or/and higher pulmonary vascular resistance) in SLE results from aCL-mediated microthrombosis and microembolization. It is noteworthy that the relative risk of clinically significant pulmonary embolism in the first year after SLE diagnosis is very high, estimated at 10.2 [71].

The connection between myocardial perfusion abnormalities detected in heart perfusion scintigraphy (SPECT) and high levels of aCL of IgG class and antiß2GPI was described [68]. Such abnormalities may also be due to microthrombosis in small coronary arteries that causes permanent (rest) perfusion defects in limited myocardial areas [7]. SPECT shows myocardial perfusion defects in half of patients with SLE [72, 73], despite normal rest ECG recordings, lack of left ventricle contractility disturbances, and clinical symptoms of myocardial ischemia [7]. In the study with 380 patients with SLE [74], a high level of aCL was associated with a high risk of myocardial infarction rather than classic atherosclerotic plaques (focal necrosis arises independently of atherosclerotic plaques but is a base for intravascular thrombosis). The non-atherosclerotic pathogenesis of myocardial ischemia explains why calcified atherosclerotic plaques are detected in SLE much less frequently than perfusion defects.

## Pharmacotherapy of endothelial dysfunction in SLE

Statins (hydroxymethylglutaryl-coenzyme A reductase [HMG-CoA] inhibitors) reveal an anti-atherosclerotic action by lowering total cholesterol, LDL cholesterol, and triglycerides and increasing high-density lipoprotein cholesterol (HDL). However, especially in connective tissue diseases, the more interesting action of statins manifests itself is an anti-inflammatory and immunomodulatory action. Statins decrease the expression of adhesion molecules on leucocytes and endothelial cells (ICAM-1, macrophage-1 antigen [MAC-1], lymphocyte function-associated antigen 1 [LFA-1]), decrease inducible expression of class II major histocompatibility complex (MHC) antigens on macrophages and other cells, lower expression of receptors for cytokines produced by Th1 lymphocytes, leading to decreased activity of T lymphocytes, and decrease their infiltration into inflammatory tissues. Additionally, statins block the synthesis of inducible nitric oxide and decrease the synthesis of pro-inflammatory cytokines (Il-6, TNFα, IFNγ), and, as a consequence, decrease the synthesis of CRP [75–78].

The beneficial immunomodulatory effect of statins has been proven in RA [79]. However, the first data on statins in SLE were controversial. Atorvastatin has been shown to reduce the progression of atherosclerosis, decrease proteinuria, and lower anti-dsDNA titers in mice [80]. Lower proteinuria after statins was also shown in humans [81]. Statins in RA decrease arterial stiffness [82, 83], improve endothelial function [84], and decrease disease activity measured by the DAS28 score [79]. On the other hand, simvastatin has been shown to produce lupus-like syndrome [85], and atorvastatin has been shown to produce dermato-polymyositis [86].

Two randomized, placebo-controlled studies on the role of statins in SLE-induced atherosclerosis were conducted. In the first study [16], atorvastatin (40 mg/day) was shown to significantly lower CRP and reduce coronary atherosclerotic plaque volume, while the volume of coronary plaques significantly increased in the 1-year observation in the placebo group. A 1-year increase in coronary calcium score in the placebo group was 85.4% [16]. It should be noted that in a large trial with 3745 participants, a lower CRP obtained during statin treatment was associated with a better prognosis, independent of the LDL cholesterol level [87]. In The Lupus Atherosclerosis Prevention Study (LAPS) [88], 40 mg/day atorvastatin slowed not significantly atherosclerosis progression, but CRP decreased in the placebo group, more than in the atorvastatin group, which may be a crucial confounder.

The limitation of statin treatment is the risk of lupus-like syndromes [85, 90, 91]. Skin changes are similar to those present in subacute cutaneous lupus. Two pathogenic mechanisms are suggested. At first, statins may aggravate cell apoptosis and leakage of nuclear antigens may lead to higher autoantibody production [92]. This is the mechanism of action of environmental factors, for example, ultraviolet rays. Second, statins may directly influence T lymphocytes, changing the balance between Th1 and Th2 on the Th2 side, increasing the response of B lymphocytes and overproduction of autoantibodies [93]. However, the risk of post-statin lupus-like syndrome is low [16, 88].

Prophylactic anticoagulation is not recommended in patients diagnosed with antiphospholipid syndrome without thromboembolic episodes, despite the level of antiphospholipid antibodies. According to the guidelines, anticoagulation is required in patients after thrombotic events [94]. Atherosclerotic plaques in the coronary arteries, SPECT-detected myocardial perfusion defects, or elevated right ventricle systolic pressure, often seen in patients with connective tissue disease [7, 68], are risk factors for death [95, 96]. The possible microthrombotic pathogenesis of these complications in autoimmune diseases directs the researcher’ attention to thrombosis prophylaxis in asymptomatic patients with high levels of antiphospholipid antibodies. In asymptomatic patients, aspirin or low molecular weight heparin was shown to efficiently reduce the number of thromboembolic complications in periods of higher risk (surgery, immobilization) [97]. Prophylaxis with aspirin and hydroxychloroquine was also efficient [98].

## SARS-CoV-2 infection, endothelial dysfunction, and thromboembolism

The main cause of death from acute Covid-19 infection is adult respiratory distress syndrome (ARDS) and thromboembolic complications, despite steroid treatment and anticoagulation [99–102]. In laboratory assessment, the acute phase of SARS-Cov-2 infection is characterized by hypercoagulable and hypofibrinolitic state (manifested mainly by high levels of D-dimers, fibrinogen, factor VIII, vWF, and high thrombin generation) [103, 104] together with the hyperinflammatory state (manifested for example, by high levels of interleukin [Il] 6 and 10, granulocyte–macrophage colony-stimulating factor and TNFa) [105].

The National Institute of Health and Care Excellence (NICE) defines the long-Covid or post-Covid syndrome as “signs and symptoms that develop during or after an infection consistent with Covid-19 and persist for more than 12 weeks and are not explained by an alternative diagnosis” [106]. In the study of nearly 50,000 people hospitalized in the UK for Covid-19 infection and discharged alive, the half-year frequency of hospital readmission was 29.4%, and 12.3% of the patients died after discharge [89]. Hospital readmissions were 3.5 times higher and deaths were 7.7 times higher than those of matched controls [107]. In the study of hospitalized Covid-19 infected patients in the USA, 20% were readmitted and 9% died within 60 days after discharge [108]. In the study of 767 patients who survived acute Covid-19 infection in Bergamo, 6% had a pulmonary embolism or deep vein thrombosis during the first 81 days after discharge [109].

In a study of 150 post-Covid patients, sustained elevation in D-dimers was a common finding after infection for up to 4 months (25.3% of patients) and occurred more frequently in those with severe acute diseases [110]. This was observed despite normalization of prothrombin time, activated partial thromboplastin clotting time, and the lack of evidence of hypofibrinogenemia or thrombocytopenia [110]. In 384 patients followed for a median of 54 days after discharge, 30% had elevated D-dimer [107]. It is speculated that post-Covid thromboembolic complications may be immunothrombotic consequences of recent infection [111]. In the study of 30 patients after Covid-19 observed up to 90 days after infection, compared to non-Covid subjects with or without cardiovascular risk factors [112], the counts of circulating endothelial cells increased significantly compared to non-Covid subjects without cardiovascular risk factors. In the same study, the levels of ICAM and pro-inflammatory cytokines (Il-1β, Il-17A, Il-2, Regulated on Activation, Normal T Cell Expressed and Secreted [RANTES]) remained elevated after Covid-19 infection. The authors state that Il-17A, Il-8, and Il-18 activate endothelial cells during atherogenesis, and their elevated levels may suggest chronic development of atherosclerotic plaques in post-Covid patients. SARS-CoV-2 has been shown to predispose to systemic autoimmunity. Reactive arthritis and connective tissue disorders such as lupus and inflammatory myositis have been reported after COVID-19 [113]. Regarding the clinically evident progression of coronary atherosclerosis, major adverse cardiovascular events (MACE) were diagnosed in post-Covid patients after discharge in 126 (121 to 131) per 1000 person-years [89].

There are many possible ways in which coagulation may be stimulated and atherosclerosis progression increased in post-Covid patients. However, according to data from acute Covid-19 studies, four main pathogenetic mechanisms may be involved: (1) endothelial activation/dysfunction, (2) presence of antiphospholipid antibodies, (3) activation of the complement system, and (4) formation of neutrophil extracellular traps (NET).

### (1) Endothelial activation and dysfunction

The recruitment and activation of inflammatory cells depend on the expression of many inflammatory mediators, such as cytokines, chemokines, and adhesion molecules: ICAM-1 and VCAM-1 [114]. Tong et al. [115] showed that ICAM-1, VCAM-1, and vascular adhesion protein-1 (VAP-1) were elevated in patients with mild Covid-19 disease and increased dramatically in severe cases.

A higher number of circulating endothelial cells were described in Covid-19 patients, especially those admitted to the intensive care unit. Their level was positively correlated with the soluble VCAM-1 [116]. The other study described an increase in circulating endothelial cells and a higher level of soluble ICAM-1 and sVCAM-1 [117]. In the study of 30 patients after Covid observed up to 90 days after infection, compared to non-Covid subjects with or without cardiovascular risk factors [112], the counts of circulating endothelial cells increased significantly compared to non-Covid subjects without cardiovascular risk factors. In the same study of ICAM, the levels remained elevated after Covid infection.

Activated endothelial cells are likely to release cytokines, which trigger the extrinsic coagulation pathway, suggesting that recovered patients may be susceptible to the risk of thrombotic complications [118].

Plasma vWF antigen (vWF: Ag), high molecular weight multimers, and propeptide levels of vWF (vWFpp) are established markers of endothelial injury [119, 120], markedly elevated during COVID-19 and may be crucial in endotheliitis and pulmonary microvascular occlusion in the pathogenesis of COVID-19 [121]. High molecular weight vWF multimers secreted in response to acute endothelium activation within the lungs may be directly involved in the trigger of lung microangiopathy [122]. Furthermore, the increase in the ratio of vWF antigen activity to ADAMTS13 was strongly associated with the severity of COVID-19 [123, 124].

### (2) Antiphospholipid antibodies

LA is found in approximately one in two patients with COVID-19, while the presence of aCL and aβ2GPI has been observed less frequently (mainly in the IgM subclass and low and medium titer), and in most cases, there are transient antibodies (no confirmation after 12 weeks) [125].

Furthermore, non-criteria antiphospholipid antibodies have been described in Covid-19. These include anti-phosphatidylserine (aPS), antiprothrombin (aPT), and anti-annexin V antibodies in IgG and IgM isotypes, as well as aCL and aβ2GPI in IgA. The high frequency and diversity of aPL strongly suggest that these antibodies are actively induced during acute SARS-CoV-2 infection. Antiphospholipid antibodies in COVID-19 are mainly directed against β2GPI but show an epitope specificity different from antibodies in antiphospholipid syndrome (directed against β2GPI domain one, which is strongly correlated with the risk of thrombosis) [126, 127]. Moreover, aPLs are not necessarily associated with thrombosis, especially if they are not persistent over time.

The question is: Are these aPLs associated with the development of vascular thrombosis, or are at least these antibodies present in a specific clinical setting? Transitory aPLs are likely to be clinically irrelevant in patients with COVID-19, as in other infections, but detecting aPLs may help identify patients potentially at risk of thrombosis.

### (3) Complement system activation

Complement system hyperactivation has been proposed as a potential driver of adverse outcomes in Covid-19 patients, given previous research of complement deposits found in tissue and blood samples and evidence of clinical improvement with anticomplement therapy [128].

Complement C3 activation products (C3a, C3b, iC3b, C3c, and C3dg) were observed in the lung even 1 day after SARS-CoV-2 infection [129]. Furthermore, C5a and soluble C5b-9 that cause endothelium damage are elevated during infection [129].

It is tempting to speculate that complement has a positive effect during the first week of infection and then (2–3 weeks of infection and in long-Covid) could induce critical hypercoagulation and hyperinflammation.

### (4) Formation of extracellular neutrophil traps (NET)

Complement activation through C3a and C5a induces the recruitment and activation of neutrophils, monocytes, eosinophils, and NETs. NETs are beneficial in host defense against viruses, but sustained NET formation—as seen in Covid-19 can trigger a cascade of inflammatory reactions that damage tissues and may enhance atherosclerotic plaque formation [129]. Complement activation in conjunction with neutrophilia and dysregulated NET formation is linked to ARDS, pulmonary inflammation, and thrombotic events. NETs initiate arterial and venous thrombosis by activating the contact pathway of coagulation, resulting in excessive generation of thrombin and C5a [130].

Elevated NET-specific markers, myeloperoxidase DNA and citrullinated histone H3, were found in infected patients [131].

## Conclusions

Endothelial dysfunction related to general inflammation in SLE creates the basis for the onset and progression of atherosclerosis and vascular thrombosis. Autoimmunity contributes to the early development of atherosclerotic plaques, myocardial ischemia, and thromboembolic complications. The acute phase of SARS-Cov-2 infection is characterized by hyperinflammatory, hypercoagulable, and hypofibrinolitic states. Patients with SLE and Covid-19 share similarities in endothelial activation/dysfunction, presence of antiphospholipid antibodies, activation of the complement system, and formation of extracellular neutrophil traps. This article presents our perspective on mechanisms underlying SLE and Covid-19, particularly endothelial dysfunction.

## Data Availability

All data generated or analysed during this study (clinical images- Figure 1-4) are included in this published article.
